# Unsolved matters in leprosy: a descriptive review and call for further research

**DOI:** 10.1186/s12941-016-0149-x

**Published:** 2016-05-21

**Authors:** Carlos Franco-Paredes, Alfonso J. Rodriguez-Morales

**Affiliations:** Infectious Diseases Clinic, Phoebe Putney Memorial Hospital, 507 3rd Avenue, Albany, GA 31721 USA; Hospital Infantil de México, Federico Gómez, Mexico D.F., Mexico; Public Health and Infection Research Group, Faculty of Health Sciences, Universidad Tecnológica de Pereira, Pereira, Risaralda Colombia

**Keywords:** Leprosy, *Mycobacterium leprae*, Hansen’s disease, Sequelae, Peripheral nerve, Schwann cell, Histiocytes, Leprosy reactions, Disability, Elimination

## Abstract

Leprosy, a chronic mycobacterial infection caused by *Mycobacterium leprae*, is an infectious disease that has ravaged human societies throughout millennia. This ancestral pathogen causes disfiguring cutaneous lesions, peripheral nerve injury, ostearticular deformity, limb loss and dysfunction, blindness and stigma. Despite ongoing efforts in interrupting leprosy transmission, large numbers of new cases are persistently identified in many endemic areas. Moreover, at the time of diagnosis, most newly identified cases have considerable neurologic disability. Many challenges remain in our understanding of the epidemiology of leprosy including: (a) the precise mode and route of transmission; (b) the socioeconomic, environmental, and behavioral factors that promote its transmission; and (c) strategies to achieve early diagnosis and prevent neurologic impairment to reduce the large burden of disability among newly identified cases; and among those who endure long-term disability in spite of completing multidrug therapy.

## Background

Leprosy is a chronic mycobacterial infection caused by *Mycobacterium leprae* leading to a plethora of clinical manifestations ranging from cutaneous manifestations to disfigurement, deformity, stigma, and disability (neurologic and blindness). The burden of disease associated with *M. leprae* infection in humans stems from the ability of this bacterial pathogen to induce severe injury of peripheral nerves (Schwann cells) and skin (keratinocytes and histiocytes) [1–7]. The clinical spectrum of disease of leprosy is further defined by the immune response to the leprosy bacillus ranging from tuberculoid, to borderline, and to lepromatous forms (Ridley-Jopling) [1, 2]. Once the infection is established, the occurrence of leprosy reactions, because of their inflammatory impact on the peripheral nerve, constitutes an important contributor to sensory loss and dysfunction [2, 3, 8, 9].

## Leprosy trends

Leprosy does not constitute the ancestral plague that once used to be. However, the elimination of leprosy as a public health problem as defined by the World Health Organization, has not been achieved in any meaningful and sustainable manner [6, 7]. Besides its measurable medical consequences, leprosy hampers the freedoms and capabilities of individuals and affected communities [10]; and often excludes individuals from social life due to the often associated stigma [11–13]. The early tales of fear and pity that leprosy in its severe forms elicited among many human groups, continues to transpire to a similar degree into modern societies [6, 7, 13].

Leprosy continues to be an important infectious disease in many endemic settings as demonstrated by: (a) a growing number of new cases [7, 14, 15]; (b) many patients completing multi-drug therapy but subsequently developing leprosy reactions [16, 17]; or (c) microbiologically treated individuals but with long-term neurologic dysfunction and disability originated by irreversible peripheral nerve injury [2, 16].

Since 1981, multi-drug therapy (MDT) has been universally instituted through active case finding in highly affected communities [6, 14]. These programs have achieved some degree of success by decreasing the prevalence of the disease [14], however, there are many continuing challenges including: (a) yearly, new cases continue to be detected in highly endemic areas [7]; (b) since 2005, the number of reported new cases has remained consistently stable despite continuous use of MDT concomitantly with a substantial decrease in the prevalence of the disease [7]; (c) a rising number of new cases are expected to reach the 4 million mark by the year 2020 [7]; and (d) From 2007 to 2013, new cases continue to be identified with grade 2 disability with no evidence of this indicator decreasing [7].

There are two major potential reasons for the persistent detection of new cases of leprosy in endemic areas. The first one is that the “elimination phase” has transitioned into an era of complacency [6, 7, 14]. The reported rate of new case detections suggests that the rate of new cases decreased by 60 % from 2000 to 2005 [7]. However, there is evidence to suggest that the detection of cases did not truly decreased to such degree during this period; and that current reports may actually represent an underestimation of newly detected cases [7]. Secondly, persistent transmission of *M. leprae* calls for reassessing our long-held notions about its mechanism and routes of transmission [18–20]. Current epidemiologic trends reinforce old disagreements regarding the portal of entry and the pathways of *M. leprae* into the human body [19, 20]. Neither person-to-person transmission nor host-susceptibility explains the patchy distribution of leprosy, and new cases are detected in persons who have had no know contact with human leprosy (30–60 % of cases) [5, 6]. Transmission of leprosy to close contacts has been documented and it is considered a major risk factor for developing leprosy among susceptible individuals [21–23]. Nonetheless, the precise mode and route of transmission has not been satisfactorily defined [22, 23]. It has been assumed that person-to-person transmission occurs by nasal secretions or cutaneous lesions under circumstances such as overcrowding, inadequate housing and lack of hygiene [21–23].

There is sufficient ecological data to suggest that the transmission of leprosy is potentially influenced by environmental factors such as soil and water exposures, insect vectors playing a role [24–35], and the free-living amoebas (e.g., *Acanthamoeba* spp.) may participate in the environmental viability of leprosy in some biotopes [30, 31]. Zoonotic transmission from natural infection of armadillos in the Southeast United States has been confirmed as responsible for the majority of autochthonous transmission of cases in this area [32]. It is likely that armadillos may also play an important role in the transmission of leprosy in some areas of Latin America such as in Colombia, Venezuela, Mexico, and Brazil [33]. Understanding how environmental factors influence host-pathogen interactions in complex natural systems [34, 35], where multiple feedbacks between biotic and abiotic factors take place, is especially important in the context of environmentally persistent pathogens such as *M. leprae*.

## Human migration and the spread of *Mycobacterium leprae*

The mycobacterial ancestor of *M. leprae* diverged from the tuberculosis bacilli approximately 66 million years ago, long before the origin of the *Homo* genus [36–42]. Estimates of the intracellular adaptation of *M. leprae* related to reductive evolution and pseudogene development has been estimated to occur around 9 million years ago [37, 38]. Our current understanding based on recent genetic and molecular clock data indicates that leprosy the human species prior or during the Paleolithic [37]. In turn, human migration has been crucial in the global spread of leprosy [38]. In this evolutionary journey, *M. leprae* has migrated with human populations through expeditionary, military, colonialist, and other human endeavours [38–41]. The earliest clinical descriptions of leprosy are said to be from Egypt and India from records dating back to 600 B.C.E [38]. Older descriptions of disfiguring cutaneous illnesses possibly including leprosy under the Hebrew Tsar’ath (zarath) contained in the Old Testament; however, this fact remains controversial since there is scant skeletal evidence for leprosy in human remains from Israel [13]. Humans are natural reservoirs in the transmission of *M. leprae* and therefore the global spread of leprosy is tied to historical milestones of human migration [10, 37–39]. Recent comparative genomic evidence points to the origin of the leprosy in Eastern Africa [38, 39]. The cohabitation of *M leprae* with human hosts has provided *M. leprae* with different social and biological attributes that facilitated the selection of different traits conferring different adaptive biological properties [37–39]. Phyleogeographic studies have demonstrated an association between the spread of leprosy and migration patterns of earlier human societies and trade routes (i.e., the Silk Road that united Europe to China contributed to the spread of leprosy) [38, 39, 41, 43]; or to historical events corresponding to the returning expeditionary forces of antiquity spreading the pathogen from the Middle-Eastern strain of *M. leprae* to Medieval Europe [38]. Subsequently, European explorers spread the disease westward to the New World or through the Atlantic slave route [37, 39]. Overall, genomic comparisons of ancient and modern strains of *M. leprae* remain remarkably similar, indicating it was probably improvements in social conditions that led to a substantial reduction of leprosy in Europe in the 16th Century [40, 41]. While these events indicate the crucial role of humans as reservoirs of disease and potentially transmitting to their close contacts, it is also feasible that nasal discharges or cutaneous lesions of populations migrating into previously leprosy-free biotopes may have caused a spillover of *M. leprae* into environmental niches with optimal biotic and abiotic factors that subsequently amplified the cycle of transmission of leprosy.

In modern times, it is likely that the clustering of cases of leprosy occurs among individuals living in resource-poor areas with favorable ecological niches for *M. leprae* to thrive [21–23]. In turn, the human host acquires *M. leprae* by an increased exposure to mycobacteria by their low socioeconomic standing combined with their biologic susceptibility to acquire the infection and develop the disease. In these settings, poverty operates by promoting low schooling, poor housing in often-unstructured settlements with overcrowding, lack safe water, absence of water management systems and sewage, and, as a result most experience poor hygienic practices [21–23]. Additionally, most individuals who have been diagnosed with leprosy have also experienced food shortages and malnutrition. Suffering from leprosy and other neglected tropical diseases becomes part of their biological destiny and their way of life. Therefore, it is important to consider the larger social drivers that underlie the unequal distribution of life choices of individuals living in the highest endemic areas that place them at risk of suffering from leprosy and other neglected diseases.

## Mycobacterial ecology

Humanity is irremediably imbedded in a matrix of natural and man-made ecologies of living organisms [44]. Mycobacteria are ubiquitous microorganisms that live in natural waters, soils, and engineered water systems that have role in nutrient cycling. A major determinant of the ecology and epidemiology of mycobacterial species is the presence of a lipid-rich outer membrane leading to biofilm formation, antibiotic/disinfectant resistance, aerosolization, and surface adherence [20, 44]. A few species have evolved from this environmental pools to become major human pathogens such as *M. tuberculosis, M. leprae* and *M. ulcerans* [13, 45–49]. Searching for common ecological patterns and transmission dynamics among these three closely phylogenetically related species may assist in identifying environmental sources of persistent infection [45–49]. For the two major human pathogens, *M. tuberculosis* and *M. leprae*, it is crucial to adapt to the intracellular lifestyle and to modulate the lipid metabolism of sanctuary cells [44] (Table 1). *M. tuberculosis* and *M. leprae* have evolved pathogenic mechanisms through complex evolutionary negotiations between these pathogens and their hosts, while the acquisition of a large plasmid encoding the toxin mycolactone relates to the underlying mechanism of pathogenicity of *M. ulcerans* [48–50]. This mycobacterial pathogen is causative agent of Buruli ulcer, which is a chronic destructive necrotizing infection of subcutaneous tissue that has been reported to occur in more than 30 countries [48–50]. In contrast to *M. tuberculosis* and *M. leprae*, *M. ulcerans* adaptation mechanisms have involved the selection of certain genes that facilitate its livelihood occupying aquatic aerobic, dark, and osmotically stable environments and its ability to reside in the extracellular matrix of the subcutaneous tissues where it unleashes the production of its toxin [50]. Genetic analyses of *M. ulcerans* have shown that it had a common ancestor with *M. marinum* and that it diverged around a million years ago [45, 49, 50]. *M. marinum* produces a relatively milder nodular cutaneous lesions compared with Buruli ulcer [50].

**Table 1:** Comparison of the three major pathogenic species within the *Mycobacterium* genus

Features	*M. tuberculosis* ^a^	*M. leprae*	*M. ulcerans* ^b^
Genome	Genome of 4.4 Mb with 4000 genes and only six pseudogenes (91 % coding capacity) No evidence of major reductive evolution	Reductive evolution and large pseudogene (1116) formation resulting in a 3.31-Mb genome Only 50 % coding capacity by 1605 genes	*M. ulcerans* downsized from 6.6 MB (*M. marinum*) to 5.8 Mb genome *M. marinum* and *M. ulcerans* share 97 % of genes since they share a common ancestor and evolved from *M.* *marinum* by lateral gene transfer and reductive evolution (~771 pseudogenes) Selected genes facilitate occupying an aerobic niche environment, osmotically stable, dark, and extracellular environments
Target cell of infection	*Intracellular* Macrophages (alveolar) and in the reticuloendothelial system Non-classical immune cells (epithelial cells, endothelial cells, fibroblasts, adipocytes, glia and neurons) [70]	*Intracellular* Schwann cells Histiocytes Keratinocytes	*Extracellular* Extracellular matrix in subcutaneous tissues where *M. ulcerans* directs the production of the polyketide toxin mycolactone^c^ leading to tissue necrosis and local tissue and systemic immunosuppression
Pathogenic mechanisms	Intracellular persistence in macrophages causes necrotizing granulomas that cause tissue destruction in the lung or other organs	Infection of Schwann cells leads to peripheral nerve dysfunction secondary to demyelination Reprogramming of Schwann cells linked to disseminated disease	Destructive ulcerative ulcers (Buruli ulcer) with subcutaneous fat necrosis
Environmental determinants	Members of the *Mycobacterium tuberculosis* complex can be shed by infected hosts to the environment in sputum, feces, and urine by humans; in milk from dairy animals (cattle) and infected body tissues from other domestic and wild animals Free-living amoebas may act as macrophage-like niches in the environment	Clustering of cases by geospatial assessments of thermal-hydrological determinants in Ethiopia and India Armadillo is a natural host in the Western Hemisphere and causes a zoonosis in the Southern USA In some settings some primates species may become reservoirs of *M. leprae* There is evidence of viable *M. leprae* found in soil, water, and free-living amoebas (*Acanthamboeba* spp.) Sphagnum and other moss vegetation may have facilitated transmission in the second half o the 19th and the beginning of the 20th century living in isolated farms in coastal Norway Ecological studies have linked contaminated water with *M. leprae* in polluted surface water in the tropics *M. leprae* contaminates clothes by washing in streams and incomplete drying in the humid tropical climate; or by bathing and washing clothes or dishes in a report from Indonesia In Brazil, bathing twice weekly, infrequent changing of bed linens or hammock among impoverished populations in Northern Brazil	*Mycobacterium ulcerans* transmission cycle involves aquatic insect vectors, aquatic plants, and aquatic animals Aquatic plants (e.g., *Rhizoclonium* sp., *H. reticulatum*) favor the growth and biofilm production of *M. ulcerans* There are two different ways that *M. ulcerans* persists in the environment and infect aquatic animals (in the savanna landscape residing in stagnant or slowly moving waters; and in tropical rainforests dwelling in alkaline waters and its growth is dependent on climate changes)
Vector transmission	*M. canettii* the ancestor of *M. tuberculosis* complex (MTC) was probably transmitted by an unidentified ancestral vector (e.g., plant or insect) prior to the Neolithic revolution (>12,000 years ago)^d^	Some mosquitoes^e^ (*Culex fatigans* and *Cimex hemipterus* caught in the field in an endemic area in India harbor viable *M. leprae* Laboratory-bred *Culex fatigans* and *Cimex hempterus* are able to take up leprosy bacilli from blood of patients with untreated lepromatous leprosy The feasibility of biting arthropods amplifying the transmission of leprosy through mechanical studies demonstrated that large numbers of bacilli are readily available to the biting apparatus of arthropods among individuals with untreated multibacillary leprosy Bacteremia in cases of leprosy may make viable bacilli available to biting arthropods	*Naucoridae* (aquatic insects) in West African Countries (Ghana, Benin, Togo)
Effect of BCG (Bacille-Calmette-Guérin)	Protection for disseminated tuberculosis including tuberculous meningitis in children <4 years of age	Variable degree of protection from different reports	Offers important protection against Buruli ulcer

Many mycobacterial species may cause skin and soft tissue infections including *M. tuberculosis, M. leprae, M. ulcerans, M. marinum, M. hemophilus, M. kansasii, M. abscessus, M. fortuitum,* and *M. chelonei* (Refs. [73])

^a^ *Mycobacterium tuberculosis* complex consists of seven species capable of causing tuberculosis (*M. canetti, M. pinnipedii, M. africae* (subtypes Ia, Ib and II), *M. microti, M. caprae, M. bovis, M. bovis* BCG, Dassie bacillus)

^b^ *Mycobacterium marinum* is a closely related species to *M. ulcerans.* Mycolatone is the toxin produced by *M. ulcerans* is responsible for causing cutaneous and subcutaneous ulceration (tissue necrosis) and associated local and systemic immunosuppression

^c^Mycolactone produced inhibition of protein translocation into the endoplasmic reticulum resulting in a deficit release of innate immune system cytokines, membrane receptors, adhesion molecules, and specific immune system cytokines

^d^Of the *Mycobacterium tuberculosis* complex, *M. bovis* and *M. caprae* are found in hosts domesticated 10,000–12,000 years ago, earlier ancestral species infected humans many years before that era and subsequently spread to other hosts directly from humans or through an unknown vector

^e^There are several biting arthropods residing in leprosy endemic areas of which any could potentially act as a vector for the transmission of leprosy

Proverbial human-to-human transmission via respiratory droplets of *M. leprae* infection has been traditionally considered the driving engine of transmission of leprosy [18, 19, 51, 52]. While leprosy bacilli are present in the nasopharynx of individuals with multibacillary leprosy [51] and from cutaneous lesions [52], and that these bacilli are able to infect other susceptible human hosts [18, 19], the precise mechanism and route of transmission remain to be completely elucidated. Indeed, the current epidemiology of the persistent transmission of leprosy along with collected evidence made since the 19th Century suggest that environmental factors such as soil and water, vegetation, arthropods [20], free-living amoebas [30, 31], and animal reservoir host such as the nine-banded armadillo (*Dasypus novemcintus*) play an influential role in the ongoing transmission of *M. leprae* [32, 33].

In 1895, Hansen and Looft made the initial observation regarding the possibility of environmental factors involved in the transmission of leprosy [24]. They suggested that the initial site of cutaneous lesions often involved sites with direct contact with environmental surfaces (e.g., wading in streams and rivers in patients with lesions in calves). Subsequently, 27 years after Hansen’s description of *M. leprae*, Sand proposed that the transmission of leprosy between humans takes place indirectly. His findings were the result of analyzing 1221 patients in the Norwegian leprosarium of Reitgjaerdet in whom the transmission within household was relatively low and most cases occurred in men who had more contact with environmental sources. He further proposed that perhaps a living organism or ground containing decomposing material were factors involved in the transmission cycle [25].

Environmental factors such as climate, type of soil and water, environmental degree of acidity [20], etc.; along with spillover of *M. leprae* from human cases (e.g., nasal discharges contaminating soil or water) may facilitate the amplification of the transmission cycle in biotopes with existing suitable ecological abiotic and biotic determinants (i.e., tropical and subtropical settings) [34, 35]. In this hypothetical model, we can postulate that chemoprophylaxis (or preemptive treatment) of contacts of multibacillary cases and effective treatment of leprosy cases decreases spillage of *M. leprae* into environmental reservoirs (soil, water, plants, or free-living amoebas) [24, 25, 27]. Preliminary evidence from a leprosy-endemic area in India has shown that genetic material of *M. leprae* was detected near washing and bathing areas where cases of leprosy were detected and genetic fingerprinting correlated between human cases and DNA detected in soil samples [24, 29]. The spillover of *M. leprae* into soil and water may explain the acquisition of this pathogen by armadillos acting as scavengers, and ultimately linking a reverse cycle of transmission from armadillos back to humans [32]. Nevertheless, it is possible that there are other unidentified environmental reservoirs or vectors influencing the occurrence of new human infections in highly endemic areas. Zoonotic transmission of *M. leprae* from armadillos in the Golf Coast of the United States contributes to endemic human infections detected in this geographic area every year, supporting the fact that leprosy is not exclusively transmitted person-to-person [32].

## Free-living amoebas as environmental sanctuaries of *M. leprae*

There is some evidence that the obligate intracellular *M. leprae* may spill over onto environmental niches and survive endosymbiotically inside free-living amoebae similar to the mechanism described of *Legionella pneumophila* residing inside *Acanthamoeba* [30, 31]. Large numbers of viable leprosy bacilli are expelled into the environment in the nasal secretions or to a lower degree from skin lesions of individuals diagnosed with multibacillary leprosy [52]. There is also evidence that *M. leprae* may invade and infect the nasal mucosa or into abraded/punctured skin [52–54]. In this regard, it is feasible that free-living pathogenic amoebae potentially act as “external” reservoirs capable of ingesting and supporting the environmental viability of *M. leprae* expelled by infectious patients into the environment and thus acting as a macrophage-like niches [30, 31]. Further evidence has demonstrated *that M. leprae* remains viable for prolonged periods inside *Acanthamoeba castellani* and *Acanthamoeba polyphaga*; and it is able to survive encystment and retain infectivity in the nu/nu mouse model [33]. It remains to be tested if *M. leprae* infected amoebae is able to transport the bacilli through nasal mucosa or through intact or abraded skin to produce clinical disease [31].

## Arthropods as vectors of *M. leprae* transmission

The possibility of arthropods as vectors of *M. leprae* has not been conclusively ruled out [5]. As early as 1915, Adolpho Lutz suggested that “*the erratic manner of the propagation of leprosy*” might be explained by the bites of biting arthropods, particularly of Culex mosquitoes (i.e., *Culex fatigans*) [20]. In fact, there are several biting arthropods residing in highly endemic areas of leprosy that theoretically might act as a vector of *M. leprae* [55–66]. In some studies, the distribution of single lesions of tuberculoid leprosy correlated with exposed skin areas [60, 61]. Mechanical studies have demonstrated the feasibility of biting arthropods to uptake *M. leprae* since large numbers of bacilli are readily available within cutaneous lesions to the biting apparatus of many species of arthropods among individuals with untreated multibacillary leprosy [59, 61–66]. Additionally, it has been shown that patients with lepromatous leprosy by developing bacteremia may make viable bacilli available to biting arthropods [63–65]. Sandflies have been ruled out as vectors of leprosy transmission [66].

There is evidence that mycobacterial species constituting the *Mycobacterium tuberculosis* complex (i.e., *M. canetti*) infected humans before the Neolithic period (< than 12,000 years ago) and that a non-mammalian vector may have played a role (e.g., plants or insects) [44–46]. Tuberculosis infection later spread to dairy animals as a result of human transmission during their domestication and involving a mechanism of transmission either through direct contact or through an unrecognized vector [44].

*Mycobacterium ulcerans* transmission cycle involves aquatic insect vectors, aquatic plants, and aquatic animals [45, 47–50]. Similarly, survival of *M leprae* in environmental niches may also involve natural reservoirs (e.g., free-living amoebas) or it may be transmitted by arthropods (e.g., mosquitoes). It is also possible that species of the *Mycobacterium tuberculosis* complex may use environmental sanctuaries such as free-living amoebas to resist the external environment by acting as a macrophage-like niche [20]. Further studies using novel molecular assays need to be conducted to assess the potential contribution of arthropods to the transmission of leprosy in endemic areas.

## Early diagnosis and neurologic disability

Peripheral nerve involvement occurs in all patients with *M. leprae* infection. At the time of a diagnosis of leprosy, up to 60 % of cases have evidence of peripheral nerve damage enough to require prolonged course of corticosteroids [6]. Neural tropism of the leprosy bacillus is through its binding and entry into Schwann cells causing demyelination [8, 9, 67–69]. These events results in demyelination of myelinated Schwann cells that manifests clinically with decreased sensorimotor function and its associated complications [69, 70]. Additionally, *M. leprae* leads to a dedifferentiation process of Schwann cells transforming them into Trojan horses for the systemic dissemination of the bacilli [3, 69, 70]. Peripheral nerve sensorimotor dysfunction in patients with leprosy is frequently exacerbated by episodes of leprosy reactions [8, 9]. Indeed, even after effective antibacterial therapy, a large number of dead bacterial cells remain within nerves and continue to elicit immunologic responses manifested as acute or chronic neuritis [8, 71]. Early detection and treatment of neuropathy in leprosy has important preventive potential. Preventing leprosy reactions or effectively treating them is therefore an important consideration in any strategy attempting to reduce peripheral nerve injury. We need to expand our understanding of factors that predispose individuals to develop leprosy reactions and the mechanisms that trigger their occurrence. One important consideration is the potential role of the microbiome in modulating the inflammatory response, particularly of herpes viruses [2]. While there is little research in this area, there is ample evidence in other clinical scenarios to illustrate that herpes viruses modulate inflammatory responses during pathologic conditions [72] (Table 2). Early identification of leprosy cases remains a central priority in controlling this disease. In this regard, school-screening programs employing clinical assessments combined with serological and molecular surveys in endemic areas have been shown to increase the early detection of cases [73]. These programs have the greatest potential for reducing transmission by early instituting of treatment early in the course of the disease; and by identifying household contacts and household cases. Similarly, geospatial analyses of risk assessments of leprosy based on thermal and hydrological environments have demonstrated useful in predicting clustering of cases in studies conducted in Ethiopia and India [34, 35] (Table 2). Efforts to scale up school-based screenings and geospatial risk assessments based on ecological determinants in hyperendemic settings may offer so far, the best opportunity to reduce the occurrence of new cases.

**Table 2:** A potential research and policy roadmap to reduce leprosy transmission

Categories	Key considerations	Suggestions
Understanding pathogenic mycobacterial ecosystems	*M. tuberculosis* complex, *M. leprae* and *M. ulcerans* are phylogenetically closely related These three pathogenic species cause three major diseases: tuberculosis, leprosy, and Buruli ulcer, respectively *Mycobacterium ulcerans* transmission cycle involves aquatic insect vectors, aquatic plants, and aquatic animals. Buruli ulcer is transmitted by aquatic fleas (*Naucoridae*) Insect vectors or plants may have played a role in much earlier transmission of tuberculosis to humans that that occurring during the neolithic revolution, where the disease spread likely from human to human and from humans to domesticated dairy animals Armadillos are responsible for the majority of autochthonous cases of human leprosy in the Southeast USA	Further studies to address zoonotic transmission by armadillos in Latin America Assess the role of armadillo control strategies to reduce leprosy transmission Potential role for household-insecticide spraying or other vector-prevention or vector-control strategies for vector control among patients diagnosed with multibacillary leprosy
Epidemiological clues linked to historical population migration events	There are important associations between the spread of leprosy to migration patterns of earlier human societies and trade routes (i.e., the Silk Road that United Europe to China contributed to the spread of leprosy); or to historical events corresponding to the returning expeditionary forces of antiquity spreading the pathogen from the Middle-Eastern strain of *M. leprae* to Medieval Europe Subsequently, European explorers spread the disease westward to the New World and through the Atlantic Slave Trade	Evaluate transmission networks Historical reassessments of important population migrations to identify potentially missed epidemiologic clues
Early diagnosis, treatment and prevention of neurologic disability	Early identification of subclinical cases may assist in interrupting the course of the natural history of the disease by preventing the occurrence of clinical manifestations including and its associated nerve injury; and from a public health perspective to potentially decrease spillage of *M. leprae* by instituting chemophrophylaxis of contacts; treatment of those with latent infection; or preemptive treatment of those with subclinical disease	Epidemiologic mapping of hot zones of transmission Rigorous contact investigation of patients with leprosy Development of a diagnostic test for early-stage or subclinical infection^a^ Early identification of leprosy cases in endemic areas through school-based surveys Implement post-exposure treatment of contacts, latent infection, or subclinical infection (chemoprophylaxis versus latent treatment versus preemptive treatment)
Preventing leprosy reactions	Leprosy reactions may occur during multi-drug therapy (MDT) or even after completing MDT Leprosy reactions are often precipitated by stress (e.g., surgery, infections, trauma) or after the initiation of MDT Herpes viruses reactive in the human host during stress Leprosy reactions exacerbate peripheral nerve injury and therefore may lead to neurologic sequelae Herpes viruses have host immunomodulatory properties and there is increasing evidence that the reactivation of some herpes viruses is responsible for drug reactions (i.e., Epstein-Barr virus (EBV) infection reaction to amoxicillin during an episode of infectious mononucleosis; or herpes human virus 6 reactivation linked to drug reaction eosinophilic systemic syndrome (HHV-6); or cytomegalovirus (CMV) causing immunologic rejection in transplant recipients	Early diagnosis of leprosy cases Close clinical follow-up of patients initiating MDT Long-term period follow up of patients that completed MDT, particularly those with borderline and lepromatous forms of leprosy Effective management of leprosy reactions to prevent further nerve injury Research to confirm the association between herpes viruses and the occurrence of leprosy reactions: evaluate patients presenting with newly diagnosed leprosy reactions with molecular testing (i.e., PCR) for herpes virus reactivation including HHV-6, Epstein-Barr virus, Varicella-Zoster virus, cytomegalovirus or others Evaluate the potential role for the institution of antiviral suppressive therapy among those with evidence of herpes virus reactivation

^a^ *Mycobacterium leprae* has the ability to reprogram the Schwann cell into a stem-cell-like cell that carries the bacilli into other tissues to ensure its dissemination. Given this systemic dissemination it seems feasible to search for the development of assays such as an interferon—assay employing a similar approach to the one used for detection of cytokine-production patterns by *M. tuberculosis*

## Conclusions

Our understanding of the transmission dynamics of *M. leprae* is incomplete. While person-to-person transmission may play a role, there is a possibility of other modes of transmission involved. Therefore, there is a need for a fresh reexamination of the historical, phyleogeographic, sociocultural, and environmental factors linked to the spread of *M. leprae* among human populations. We need to consider mycobacterial ecologies of other pathogenic mycobacteria such as *M. ulcerans;* and to expand our exploration for environmental determinants including thermal-hydrological factors (i.e., soil, vegetation, water); intermediate reservoirs or vectors including free-living amoebas, arthropods, and zoonotic transmission. Identifying epidemiologic clues from these analyses may facilitate designing effective control or elimination interventions.
